# Supplementary material for: Inhibition of base editors with anti-deaminases derived from viruses
Source: Nat Commun. 2022 Feb 1;13:597. doi: 10.1038/s41467-022-28300-0 (PMC8807840; doi:10.1038/s41467-022-28300-0)

Supplementary Information

Inhibition of base editors with anti-deaminases derived from viruses

Zhiquan Liu[1#], Siyu Chen[1#], Liangxue Lai[1,2,3,4]*, Zhanjun Li[1]*

Supplementary Figure 1. Base editing of RmA3Bctd-, mA3CDA1- and SsA3Bctd-CBE with Ades.

Supplementary Figure 2. Immunoblots of A3-CBEs in the presence or absence of corresponding Ades.

Supplementary Figure 3. Base editing of A3-CBE chimeras.

Supplementary Figure 4. Ade1 and Ade2 relocalized A3Bctd- and A3A-CBEs.

Supplementary Figure 5. *In vitro* deaminase activity assay of A3A and A3Bctd.

Supplementary Figure 6. Base editing of rA1-, eAID-, eCDA1-CBE and ABE8e with Ades.

Supplementary Figure 7. Comparison of base editing characteristics by rA1-, A3B- and A3Bctd-CBE.

Supplementary Figure 8. C-to-G base editing by rA1 variants-, A3A variants-, and A3Bctd variants-CGBEs.

Supplementary Figure 9. Evaluation of on-target and off-target editing by A3A- and A3Bctd-CBE in HEK site4.

Supplementary Figure 10. Evaluation of on-target and off-target editing by A3G-CBE in EMX1-1.

Supplementary Figure 11. Ade2 moderately narrowed the C-to-T editing window of A3A-CBE.

Supplementary Figure 12. Base editing of A3A- and A3Bctd-CBE with both Acr and Ade.

Supplementary Table 1. Target sites used in this study.

Supplementary Table 2. Primers used for genotyping in this study.

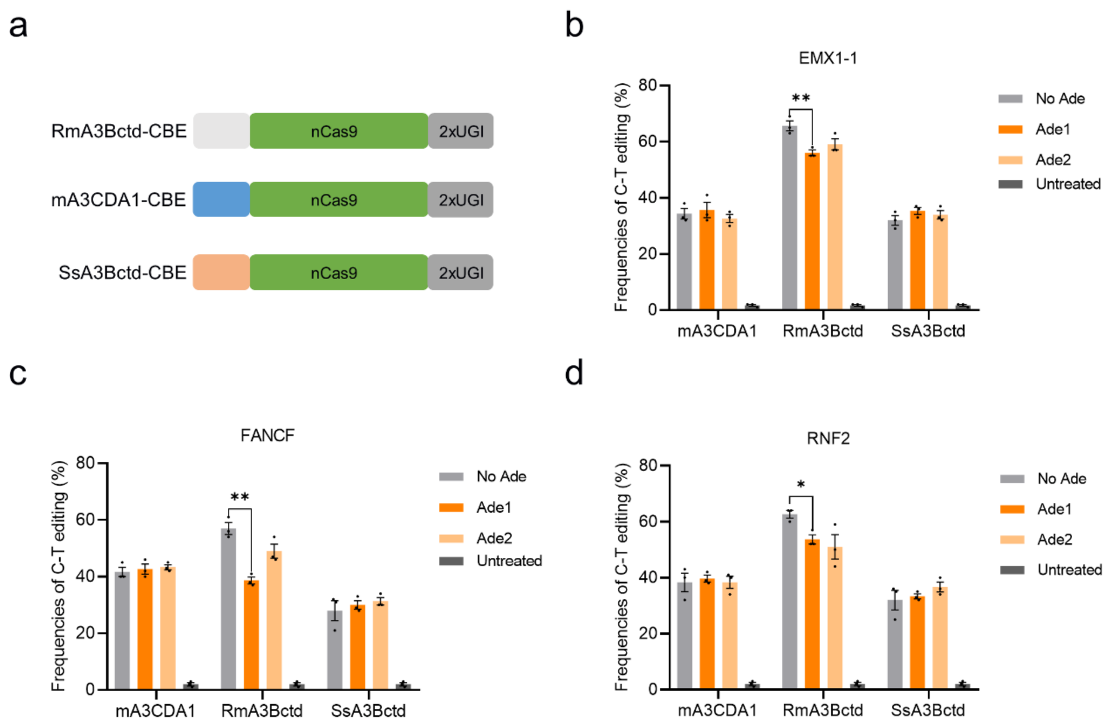

Supplementary Figure 1. Base editing of RmA3Bctd-, mA3CDA1- and SsA3Bctd-CBE with Ades. **a** Schematic representation of RmA3Bctd-, mA3CDA1- and SsA3Bctd-CBE. nCas9, D10A. **b-d** Base editing of these three CBEs in the presence or absence of the Ade1 and Ade2 at EMX1-1, FANCF and RNF2 site. Values and error bars reflect the mean ± s.e.m. and n=3 biologically independent experiments. All p values were calculated by two-sided t tests. *$p < 0.05$, **$p < 0.01$. Source data are provided as a Source Data file.

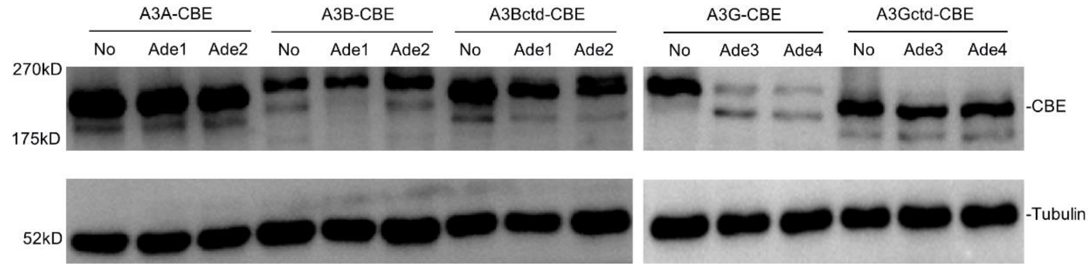

Supplementary Figure 2. Immunoblots of A3-CBEs in the presence or absence of corresponding Ades. Tubulin was used as a loading control. The western blot experiments were repeated three times independently with similar results.

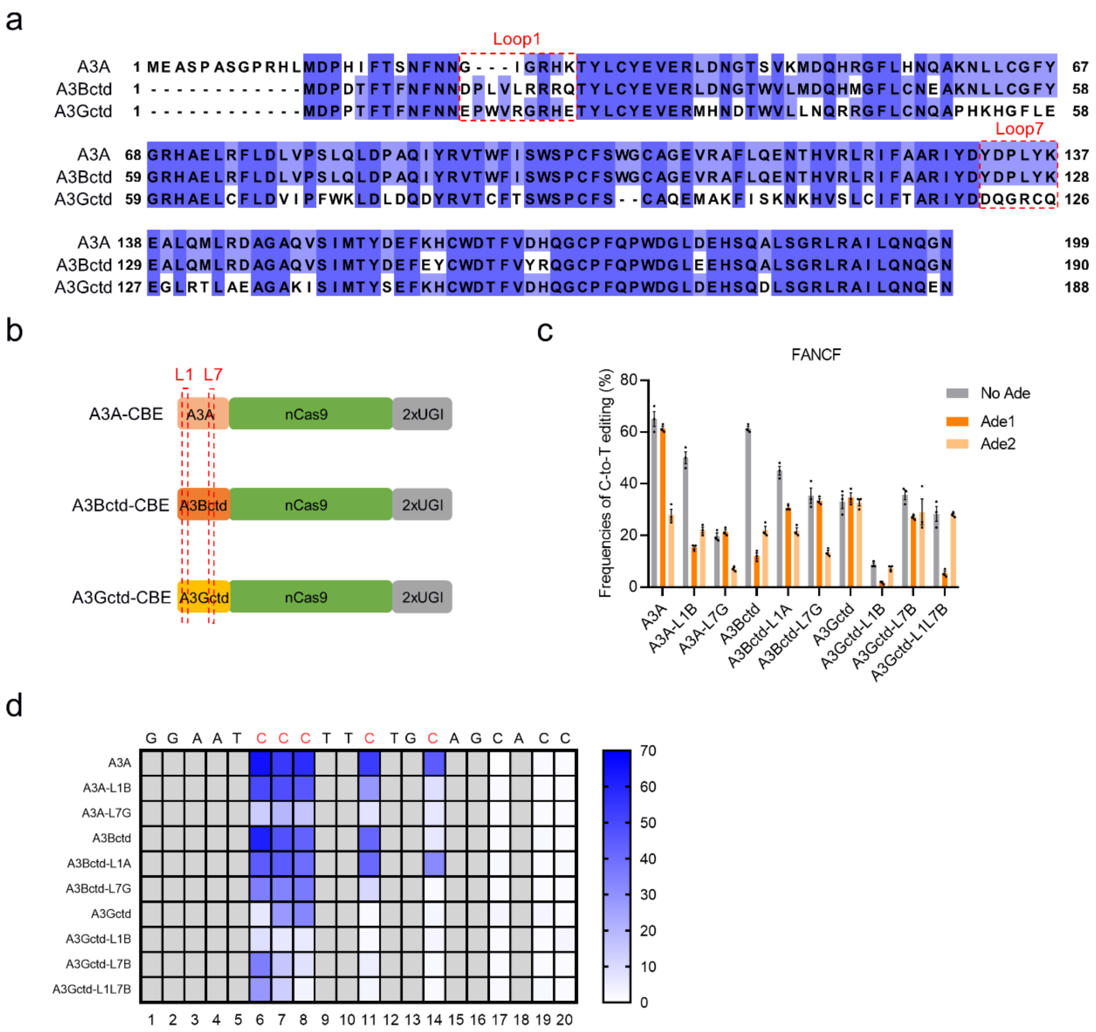

Supplementary Figure 3. Base editing of A3-CBE chimeras. **a** Amino acid sequence alignment of A3A, A3Bctd and A3Gctd. Alignment was performed with NCBI COBALT and rendered using Jalview. The L1 and L7 are shown in red rectangle. **b** Schematic representation of A3-CBE chimeras by exchanging L1 and L7 among A3A, A3Bctd and A3Gctd. nCas9, D10A. **c** Base editing of A3-CBE chimeras in the presence or absence of the Ade1 and Ade1 at FANCF site. **d** Heat map showing summary of C-to-T editing frequencies on each base for these A3-CBE chimeras at FANCF site. Values and error bars reflect the mean ± s.e.m. and n=3 biologically independent experiments. Source data are provided as a Source Data file.

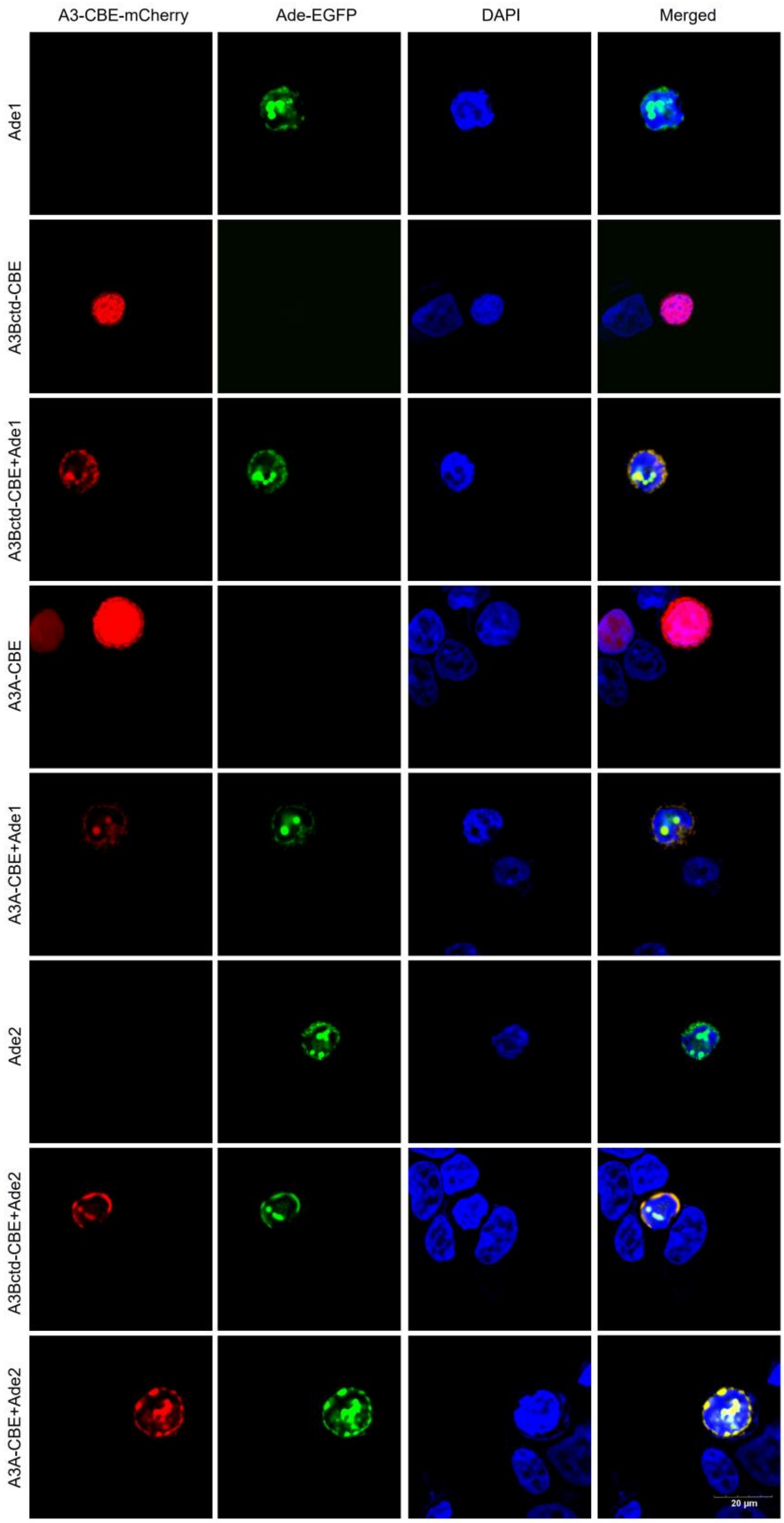

Supplementary Figure 4. Ade1 and Ade2 relocalized A3Bctd- and A3A-CBEs. Representative images of HEK293T cells expressing the indicated A3-CBE-mCherry constructs alone or in combination with Ade-EGFP constructs. The immunofluorescence microscopy experiments were repeated three times independently with similar results.

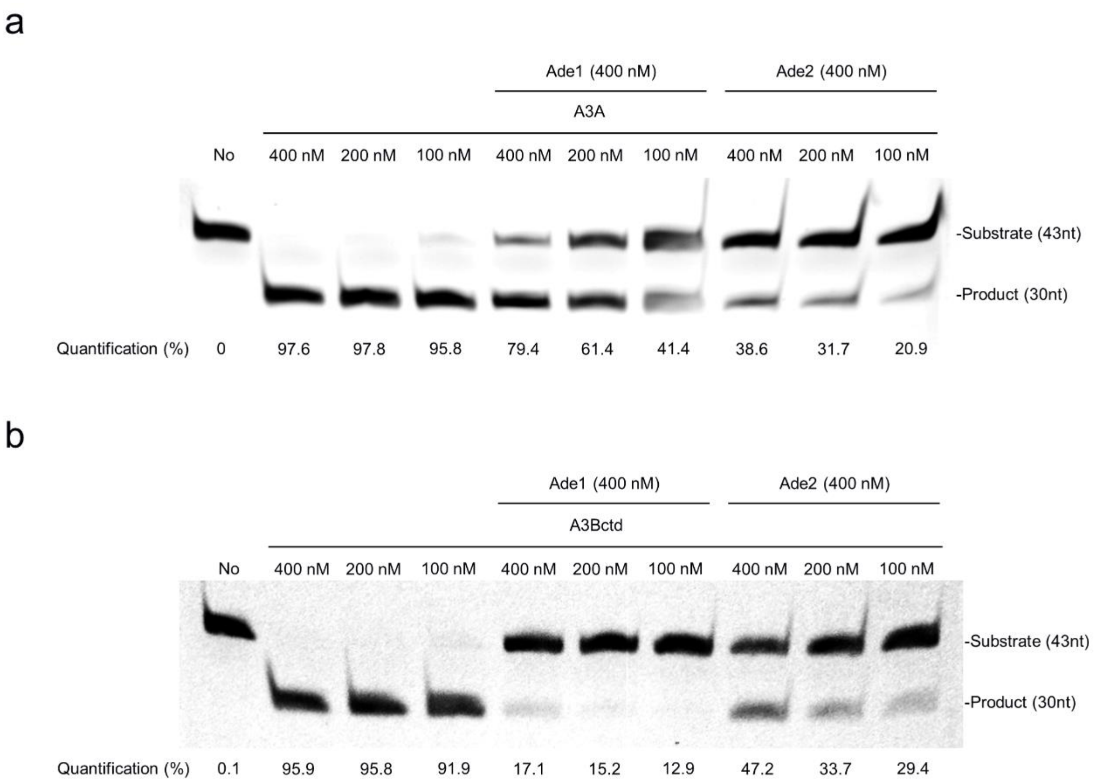

Supplementary Figure 5. *In vitro* deaminase activity assay of A3A and A3Bctd. TBE-urea PAGE analysis of A3A (**a**) and A3Bctd (**b**) deaminase activity in the presence of Ade1 or Ade2 (product percentage indicated below each lane). The experiment was repeated three times independently with similar results.

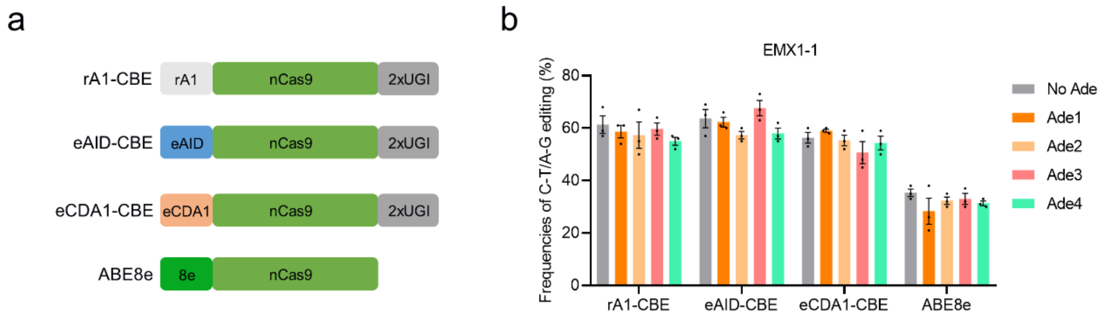

Supplementary Figure 6. Base editing of rA1-, eAID-, eCDA1-CBE and ABE8e with Ades. **a** Schematic representation of rA1-, eAID-, eCDA1-CBE and ABE8e. nCas9, D10A. **b** Base editing of these four BEs in the presence or absence of the Ade1-Ade4 at EMX1-1 site. Values and error bars reflect the mean ± s.e.m. and n=3 biologically independent experiments. Source data are provided as a Source Data file.

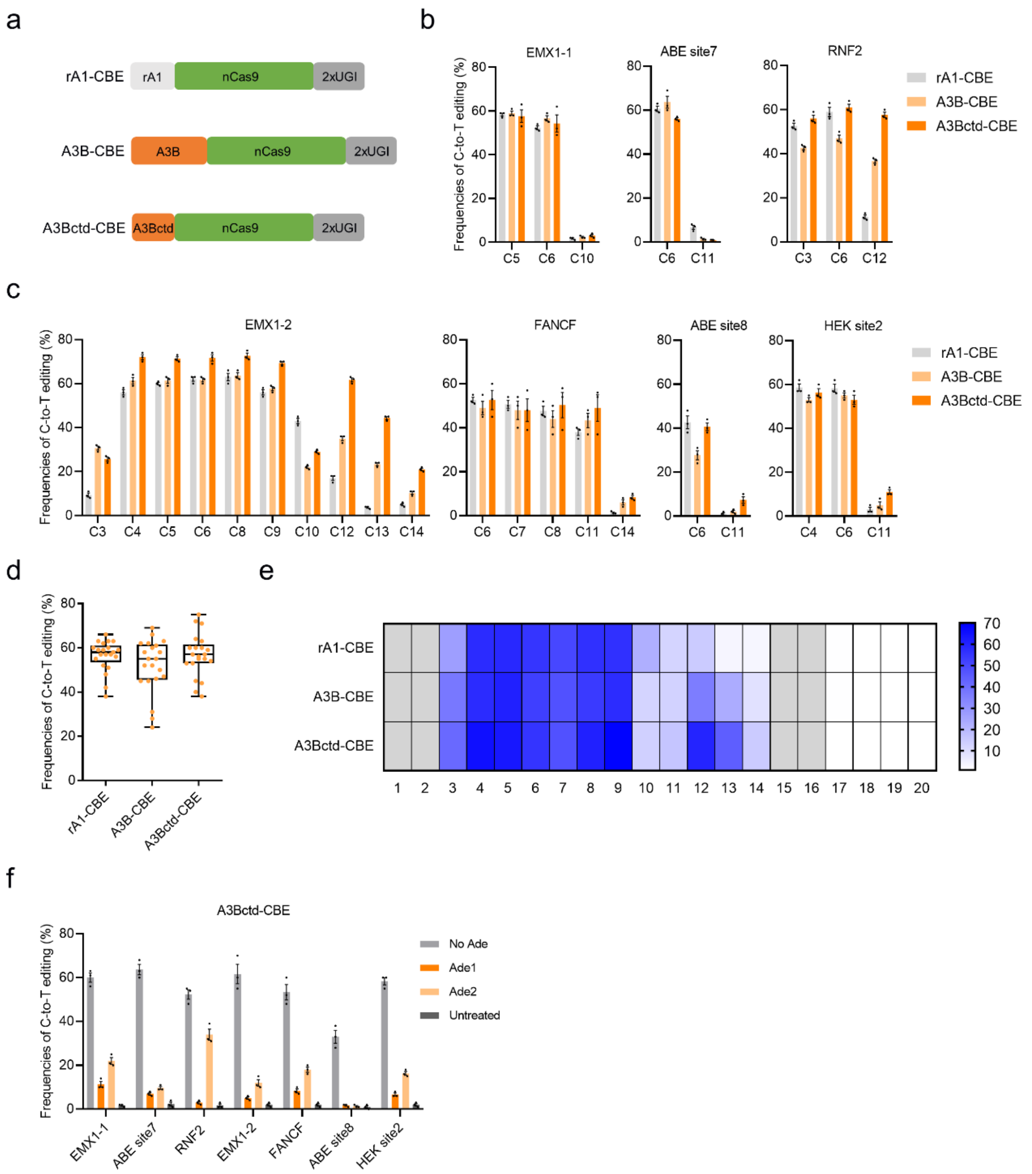

Supplementary Figure 7. Comparison of base editing characteristics by rA1-, A3B- and A3Bctd-CBE. **a** Schematic representation of rA1-, A3B- and A3Bctd-CBE. nCas9, D10A. **b-c** Summary of C-to-T editing frequencies induced by rA1-, A3B- and A3Bctd-CBE systems on each cytosine at seven target sites. **d** Summary of average C-to-T editing frequencies induced by rA1-, A3B- and A3Bctd-CBE systems at seven target sites in (**b**) and (**c**). The center line indicates the median, and the bottom and top lines of the box represent the minima and maxima, respectively. **e** Heat map showing summary of C-to-T editing frequencies on each base for rA1-, A3B- and A3Bctd-CBE. **f** Base editing of A3Bctd-CBE in the presence or absence of the Ade1 and Ade2 at seven sites. Values and error bars reflect the mean ± s.e.m. and n=3 biologically independent experiments. Source data are provided as a Source Data file.

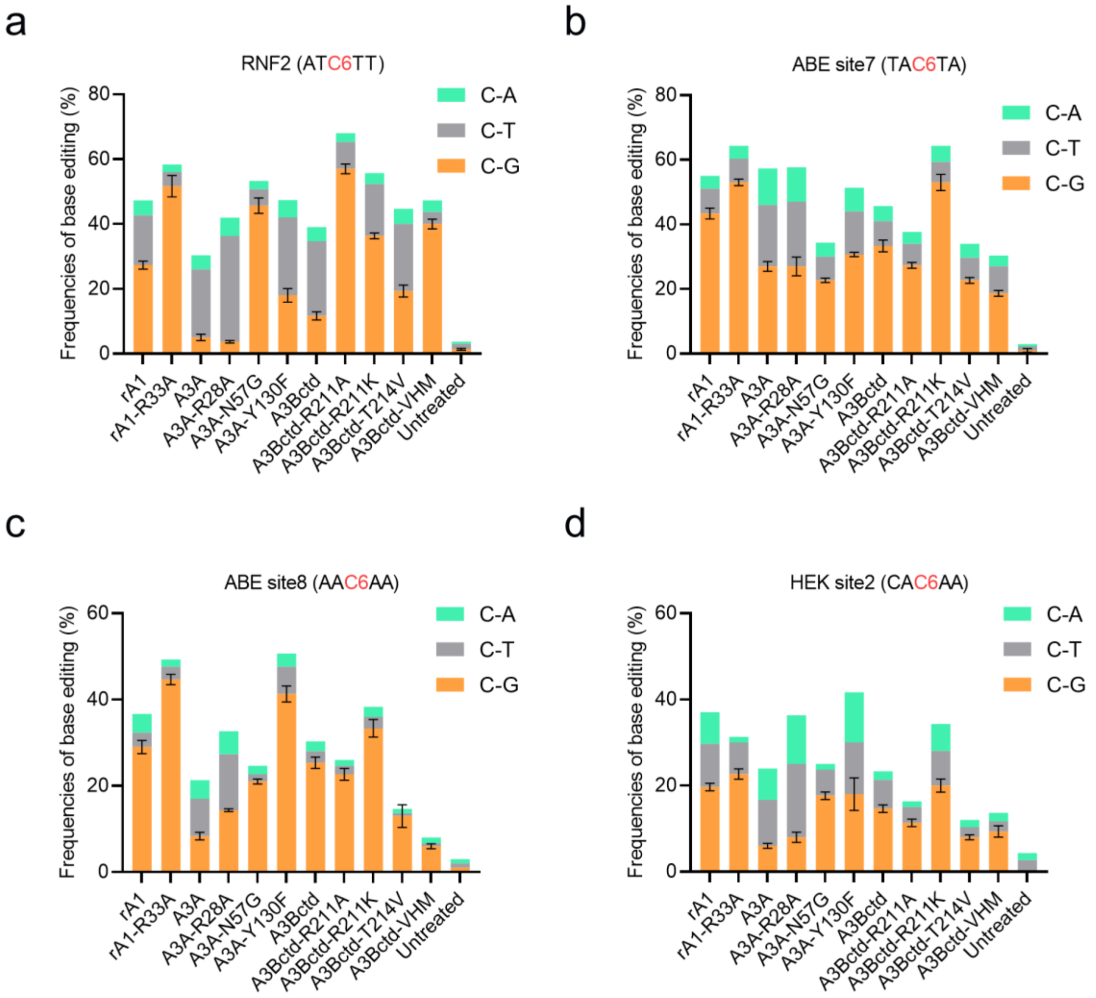

Supplementary Figure 8. C-to-G base editing by rA1 variants-, A3A variants-, and A3Bctd variants-CGBEs. **a-d** Bar plots showing the base editing frequencies induced by rA1 variants-, A3A variants-, and A3Bctd variants-CGBEs at RNF2, ABE site7, ABE site8 and HEK site2. Target C, red. Plasmids expressing CGBE, sgRNA, and Ade (1:1:1) were co-transfected into HEK293T cells. Values and error bars reflect the mean ± s.e.m. and n=3 biologically independent experiments. Source data are provided as a Source Data file.

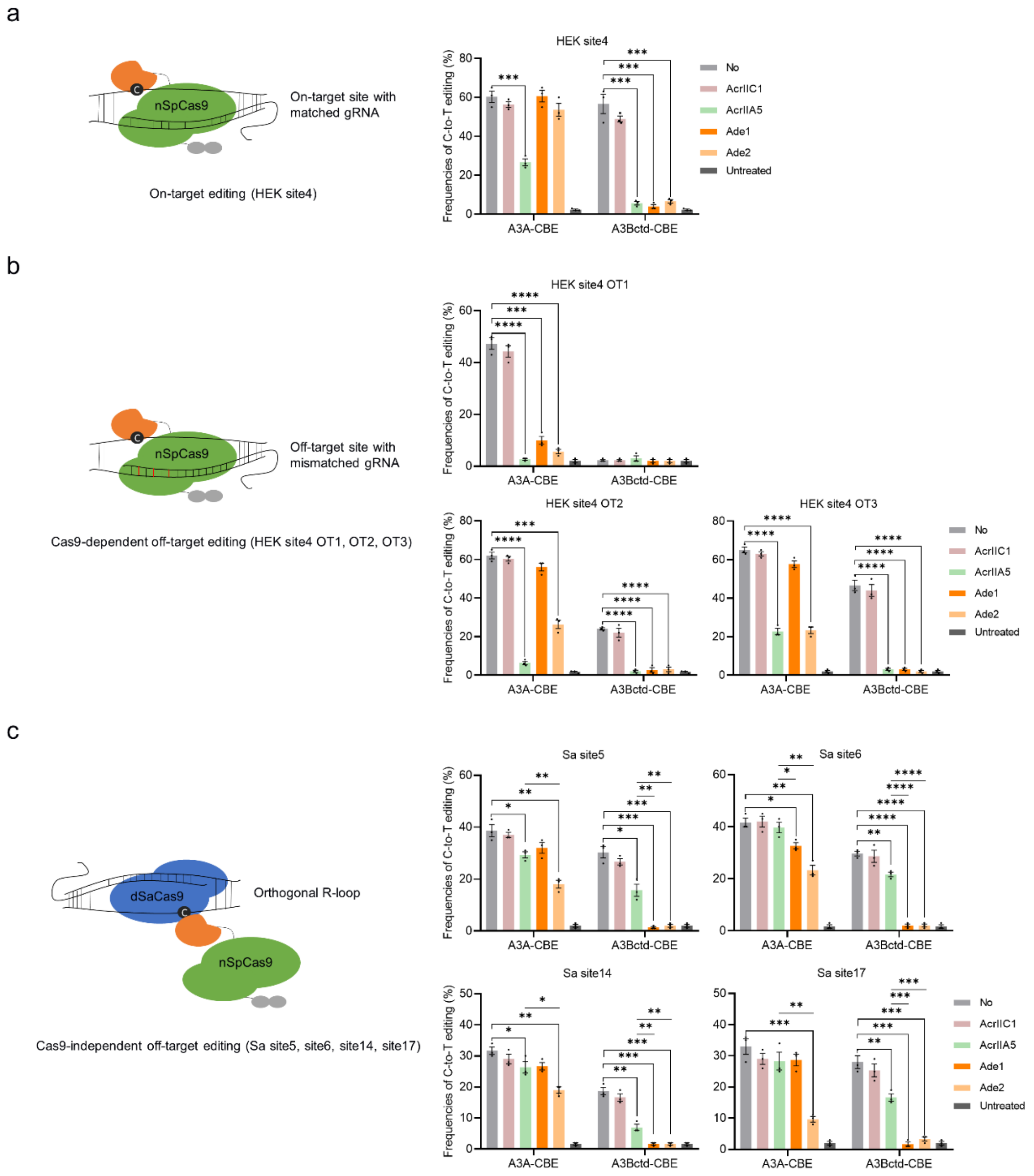

Supplementary Figure 9. Evaluation of on-target and off-target editing by A3A- and A3Bctd-CBE in HEK site4. **a** On-target editing frequencies of A3A and A3Bctd-CBE in the presence or absence of the AcrIIC1 (as a negative control), AcrIIA5, Ade1 and Ade2 at HEK site4. **b** The Cas9-dependent OT editing frequencies of A3A and A3Bctd-CBE in the presence or absence of the AcrIIC1 (as a negative control), AcrIIA5, Ade1 and Ade2 at OT1-OT3 sites. **c** The Cas9-independent OT editing frequencies of A3A and A3Bctd-CBE in the presence or absence of the AcrIIC1 (as a negative control), AcrIIA5, Ade1 and Ade2 at Sa site5, site6, site 14 and site17. Values and error bars reflect the mean ± s.e.m. and n=3 biologically independent experiments. All *p* values were calculated by two-sided t tests. *$p < 0.05$, **$p < 0.01$, ***$p < 0.001$, ****$p < 0.0001$. Source data are provided as a Source Data file.

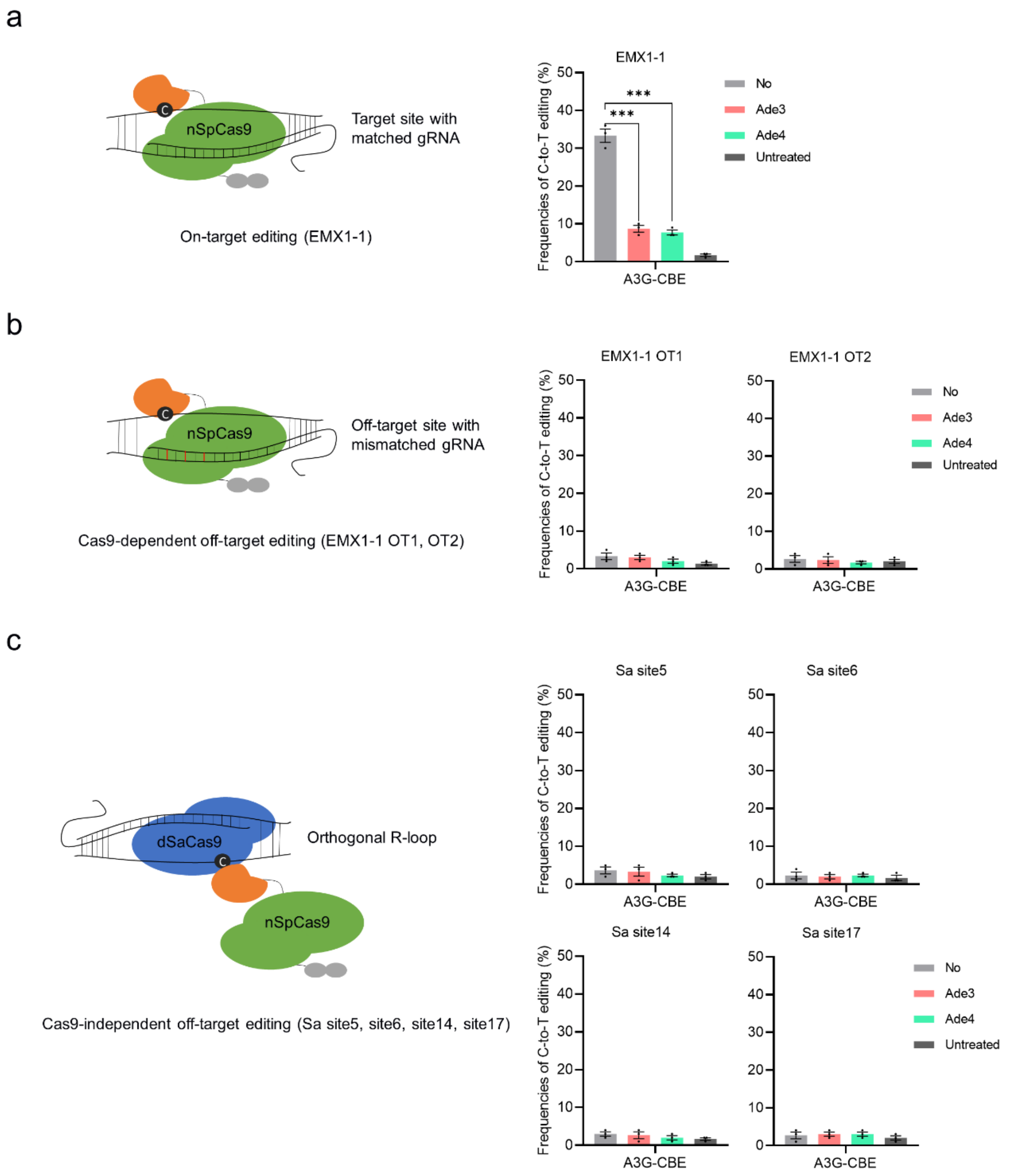

Supplementary Figure 10. Evaluation of on-target and off-target editing by A3G-CBE in EMX1-1. **a** On-target editing frequencies of A3G-CBE in the presence or absence of the Ade3 and Ade4 at EMX1-1 site. **b** The Cas9-dependent OT editing frequencies of A3G-CBE in the presence or absence of the Ade3 and Ade4 at EMX1-1 OT1 and OT2 site. **c** The Cas9-independent OT editing frequencies of A3G-CBE in the presence or absence of the Ade3 and Ade4 at Sa site5, site6, site14 and site17. Values and error bars reflect the mean ± s.e.m. and n=3 biologically independent experiments. All p values were calculated by two-sided t tests. *$p < 0.05$, **$p < 0.01$, ***$p < 0.001$, ****$p < 0.0001$. Source data are provided as a Source Data file.

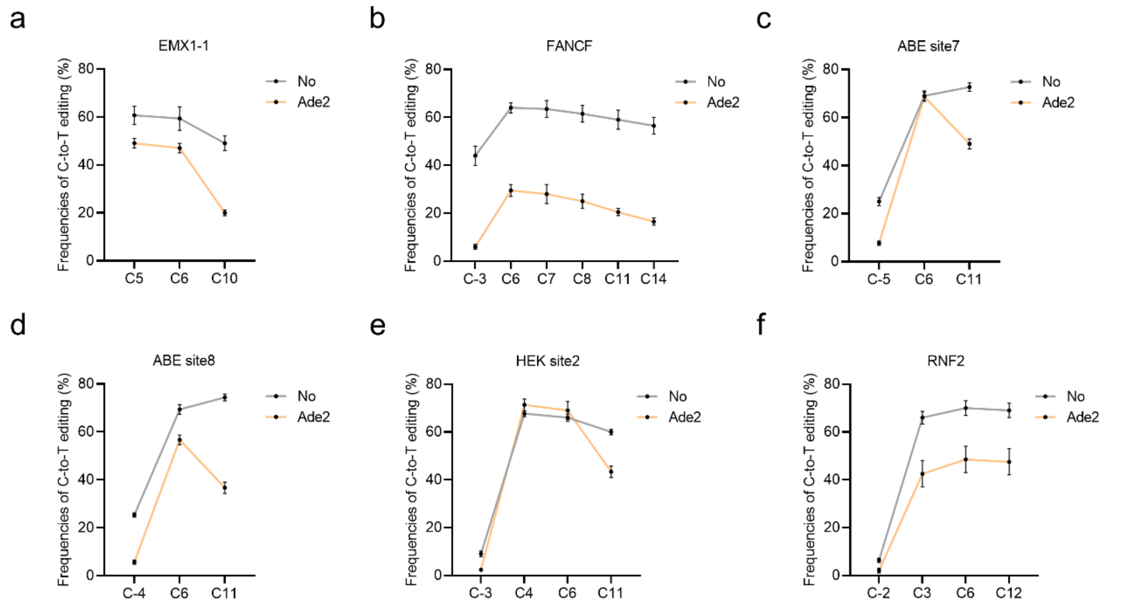

Supplementary Figure 11. Ade2 moderately narrowed the C-to-T editing window of A3A-CBE. **a-f** Frequencies of single C-to-T conversions using A3A-CBE in the presence or absence of Ade2 at six target sites. Values and error bars reflect the mean ± s.e.m. and n=3 biologically independent experiments. Source data are provided as a Source Data file.

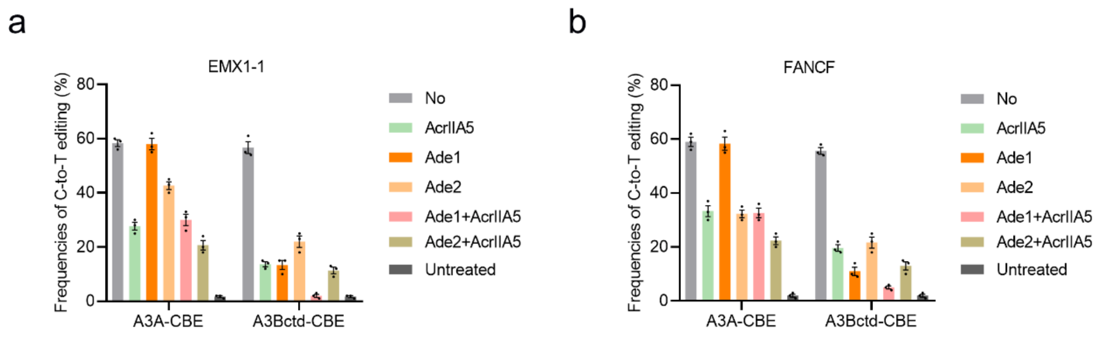

Supplementary Figure 12. Base editing of A3A- and A3Bctd-CBE with both Acr and Ade. **a-b** Base editing of A3A- and A3Bctd-CBE in the presence or absence of the AcrIIA5 alone, Ade1 alone, Ade2 alone, Ade1+AcrIIA5 and Ade2+AcrIIA5 at EMX1-1 and FANCF site. Values and error bars reflect the mean ± s.e.m. and n=3 biologically independent experiments. Source data are provided as a Source Data file.

Supplementary Table 1. Target sites used in this study. Target sequence (black), PAM region (green).

Target site	Sequence (5'-3')
EMX1-1	GAGTCCGAGCAGAAGAAGAAGGG
FANCF	GGAATCCCTTCTGCAGCACCTGG
EMX1-2	TGCCCCTCCCTCCCTGGCCCAGG
RNF2	GTCATCTTAGTCATTACCTGAGG
ABE site7	GAATACTAAGCATAGACTCCAGG
ABE site7	GTAAACAAAGCATAGACTGAGGG
HEK site2	GAACACAAAGCATAGACTGCGGG
HEK site4	GGCACTGCGGCTGGAGGTGGGGG
EMX1-1 OT1	GAGTCTAAGCAGAAGAAGAAGAG
EMX1-1 OT2	GAATCCAAGCAGAAGAAGAGAAG
HEK site4 OT1	GACACTGCAGCTGGAGGTGGGG
HEK site4 OT2	GGCACGACGGCTGGAGGTGGGGG
HEK site4 OT3	GGCATCACGGCTGGAGGTGGAGG
Sa site5	TCTGCTTCTCCAGCCCTGGCCTGGGT
Sa site6	GATGTTCCAATCAGTACGCAGAGAGT
Sa site14	GGCCTCCGTATCACTCTCTGACTGGGGT
Sa site17	GGTCGTAGCCAGTCCGAACCCCGGAGT

Supplementary Table 2. Primers used for genotyping in this study.

Primers	Sequence (5'-3')	Product size (bp)	Target sites
EMX1-F:<br>EMX1-R:	CCTTCTGTGAATGTTAGACCCA<br>TGCTTGTCCCTCTGTCAATG	520	EMX1-1,<br>EMX1-2
FANCF-F:<br>FANCF-R:	ATCTGCTCTCCCTCCACTAA<br>GGCCTGGAAGTTCGCTAAT	483	FANCF
RNF2-F:<br>RNF2-R:	GTCTTCCTTGGTGCCTTATCA<br>TGGTGGGACACATACATTCAG	524	RNF2
AS7-F:<br>AS7-R:	CATCTTCTCCGCAGACAGAAATA<br>TCCTGCCCTTAGGTCAAATAAC	465	ABE site7
AS8-F:<br>AS8-R:	CATACTTAAAGTAAAGCAGAAGGAATAACAGTGC<br>GAAGTTTCATTCCACTATAGAACTGGTTCTG	225	ABE site8
HS2-F:<br>HS2-R:	GGACGTCTGCCCAATATGTAA<br>GCATTAATCCACAGCAACACC	455	HEK site2
HS4-F:<br>HS4-R:	CCCTTCAAGATGGCTGACAA<br>TTTCAACCCGAACGGAGAC	230	HEK site4
E-OT1-F:<br>E-OT1-R:	GCTTCTTGTTCTTTGGCTTTCT<br>AGGGTATAGTTGAGGTGGGATA	476	EMX1-1 OT1
E-OT2-F:<br>E-OT2-R:	CATCTTCTGCTGGTTCCGATTA<br>GATCAGGGCGAGATTAGAGTTG	407	EMX1-1 OT2
H-OT1-F:<br>H-OT1-R:	CAGTGGCTCATCCCTGTAATTC<br>CCACACACAATCCAAACAAAGG	407	HEK site4 OT1
H-OT2-F:<br>H-OT2-R:	GGTTGCCTTTCTCCTTGGT<br>GTCCAGATTCTCTGGTGTGTG	403	HEK site4 OT2
H-OT3-F:<br>H-OT3-R:	GTATCCTGTTATCTGTTGCTGAAATG<br>CAGGTGTGGAAGAGAAATGAAATG	409	HEK site4 OT3
Sa5-F:<br>Sa5-R:	ATGTGGGCTGCCTAGAAAGG<br>CCCAGCCAAACTTGTCAACC	297	Sa site5
Sa6-F:<br>Sa6-R:	TAGGATGCCCTACATCTGCTCTC<br>CGGAAAAGCGATCCAGGTGC	278	Sa site6
Sa14-F:<br>Sa14-R:	CCCACAGACTAGCCAAGAAAC<br>CCTTCTCTTGAACCAACTCCTATAC	401	Sa site14
Sa17-F:<br>Sa17-R:	GGCTGTCTTGGGATGTTTCT<br>GGCCAGTGGAGGATCTTTATTAG	271	Sa site17

Supplementary sequence

A3A:

MEASPASGPRHLMDPHIFTSNFNNGIGRHKTYLCYEVERLDNGTSVKMDQHRGFLHNQA
KNLLCGFYGRHAELRFLDLVPSLQLDPAQIYRVTWFISWSPCFSWGCAGEVRAFLQENTH
VRLRIFAARIYDYDPLYKEALQMLRDAGAQVSIMTYDEFKHCWDTFVDHQGCPFQPWDG
LDEHSQALSGRLRAILQNQGN

A3B:

MNPQIRNPMERMYRDTFYDNFENEPILYGRSYTWLCYEVKIKRGRSNLLWDTGVFRGQV
YFKPQYHAEMCFLSWFCGNQLPAYKCFQITWFVSWTPCPDCVAKLAEFLSEHPNVTLTIS
AARLYYYWERDYRRALCRLSQAGARVKIMDYEEFAYCWENFVYNEGQQFMPWYKFDE
NYAFLHRTLKEILRYLMDPDTFTFNFNNDPLVLRRRQTYLCYEVERLDNGTWVLMDQHM
GFLCNEAKNLLCGFYGRHAELRFLDLVPSLQLDPAQIYRVTWFISWSPCFSWGCAGEVRA
FLQENTHVRLRIFAARIYDYDPLYKEALQMLRDAGAQVSIMTYDEFEYCWDTFVYRQGC
PFQPWDGLEEHSQALSGRLRAILQNQGN

A3Bctd:

MDPDTFTFNFNNDPLVLRRRQTYLCYEVERLDNGTWVLMDQHMGFLCNEAKNLLCGFY
GRHAELRFLDLVPSLQLDPAQIYRVTWFISWSPCFSWGCAGEVRAFLQENTHVRLRIFAAR
IYDYDPLYKEALQMLRDAGAQVSIMTYDEFEYCWDTFVYRQGCPFQPWDGLEEHSQALS
GRLRAILQNQGN

A3G:

MKPHFRNTVERMYRDTFSYNFYNRPILSRRNTVWLCYEVKTKGPSRPPLDAKIFRGQVYS
ELKYHPEMRFFHWFSKWRKLHRDQEYEVTWYISWSPCTKCTRDMATFLAEDPKVTLTIF
VARLYYFWDPDYQEALRSLCQKRDGPRATMKIMNYDEFQHCWSKFVYSQRELFEPWNN
LPKYYILLHIMLGEILRHSMDPPTFTFNFNNEPWVRGRHETYLCYEVERMHNDTWVLLN
QRRGFLCNQAPHKHGFLEGRHAELCFLDVIPFWKLDLDQDYRVTCFTSWSPCFSCAQEM
AKFISKNKHVSLCIFTARIYDDQGRCQEGLRTLAEAGAKISIMTYSEFKHCWDTFVDHQGC
PFQPWDGLDEHSQDLSGRLRAILQNQEN

A3Gctd:

MDPPTFTFNFNNEPWVRGRHETYLCYEVERMHNDTWVLLNQRRGFLCNQAPHKHGFLE
GRHAELCFLDVIPFWKLDLDQDYRVTCFTSWSPCFSCAQEMAKFISKNKHVSLCIFTARIY
DDQGRCQEGLRTLAEAGAKISIMTYSEFKHCWDTFVDHQGCPFQPWDGLDEHSQDLSGR
LRAILQNQEN

Ade1 (EBV-BORF2):

MATTSHVEHELLSKLIDELKVKANSDPEADVLAGRLLHRLKAESVTHTVAEYLEVFSDKF
YDEEFFQMHRDELETRVSAFAQSPAYERIVSSGYLSALRYYDTYLYVGRSGKQESVQHFY
MRLAGFCASTTCLYAGLRAALQRARPEIESDMEVFDYYFEHLTSQTVCCSTPFMRFAGVE
NSTLASCILTTPDLSSEWDVTQALYRHLGRYLFQRAGVGVGVTGAGQDGKHISLLMRMIN
SHVEYHNYGCKRPVSVAAYMEPWHSQIFKFLETKLPENHERCPGIFTGLFVPELFFKLFRD
TPWSDWYLFDPKDAGDLERLYGEEFEREYYRLVTAGKFCGRVSIKSLMFSIVNCAVKAGS
PFILLKEACNAHFWRDLQGEAMNAANLCAEVLQPSRKSVATCNLANICLPRCLVNAPLAV

RAQRADTQGDELLLALPRLSVTLPGEGAVGDGFSLARLRDATQCATFVVACSILQGSPTYD
SRDMASMGLGVQGLADVFADLGWQYTDPPSRSLNKEIFEHMYFTALCTSSLIGLHTRKIF
PGFKQSKYAGGWFHWHDWAGTDLSIPREIWSRLSERIVRDGLFNSQFIALMPTSGCAQVT
GCSDAFYPFYANASTKVTNKEEALRPNRSFWRHVRLDDREALNLVGGRVSCLPEALRQR
YLRFQTAFDYNQEDLIQMSRDRAPFVDQSQSHSLFLREEDAARASTLANLLVRSYELGLK
TIMYYCRIEKAADLGVMECKASAALSVPREEQNERSPAEQMPPRPMEPAQVAGPVDIMSK
GPGEGPGGWCVPGGLEVCYKYRQLFSEDDLLETDGFTERACESCQ

Ade2 (KSHV-ORF61):
MSVRTFCQVHLGAVPPERRGSQVSAGQLSDFDMCAQSLIDFLKVRVGWDVRANAMAGR
LWHQIMEARCPATLKQYLGIFRGVLGHRVESFIQKNIDALEDMLCAYRRSKAYEDTLNCG
YLSAVRLYDTYVLRTMGTEPVYESVAQMFMRVSVFVACQCLEHECLYWLARDLIEDAKS
VSEMAIVEYVFGYLAAQHVCCATPILRSAGVEGGQLASCFILQPSMMNEPGTLDALYHD
MSPLLASKSGVGLDVTSFSHQKNIASCLKLVDAQVHYFNDNNIRPVGASAYMELWHSQIC
DFLNAKLPENPDRCHSLFQGVCIPTLFFRMYEKDPSKLWYLFDPATAPNLIKLYGAAFDNE
YERLVRAGKYVSCMPLKSMMFTLIHTIIKTGSPYVLLKEALNEHHWTDTQGMAINCSNLC
AEIVQLPGRNTSVCNLANICLPKCLRTVESARVGTTDANRPFFCFEALGDAVRVAVLVINA
CILGGSHPTPGVERGQKERSMGIGVQGLADVFAELGYGYLDAESAELDKNIFQSMYYTAV
ETSHNLVLEGQGVPFHGWEVSNFAKGRFHWQTWEGEDASFVPRHRWDALGKSIAEHGIF
NSQFLAVMPTAGTSQVTGYAESVYPFFANISSKVTNKEEVLRPNVTFFKKVLPDDLRVVRQ
YGGDVSTFPKHHRERYRVFLTAFDYCPFKLLDRARARAPFVDQSQSMSFFLKEDRVRNAS
YLRDLLLHGYRLGLKTLMYYCRVQKQSSLTALQCLADPGSPPHSGMKQDGAWLPGPKNP
EEESCAADPECLVCQ

Ade3 (HIV-1-Vif):
MENRWQVMIVWQVDRMRIRTWKSLVKHHMYVSGKARGWFYRHHYESPHPRISSEVHIP
LGDARLVITTYWGLHTGERDWHLGQGVSIEWRKKRYSTQVDPELADQLIHLYYFDCFSD
SAIRKALLGHIVSPRCEYQAGHNKVGSLQYLALAALITPKKIKPPLPSVTKLTEDRWNKPQ
KTKGHRGSHTMNGH

Ade4 (SIVmac239-Vif):
MEEEKRWIAVPTWRIPERLERWHSLIKYLKYKTKDLQKVCYVPHFKVGWAWWTCSRVIF
PLQEGSHLEVQGYWHLTPEKGWLSTYAVRITWYSKNFWTDVTPNYADILLHSTYFPCFTA
GEVRRAIRGEQLLSCCRFPRAHKYQVPSLQYLALKVVSDVRSQGENPTWKQWRRDNRR
GLRMAKQNSRGDKQRGGKPPTKGANFPGLAKVLGILA

Ade5 (HSV-1-ICP6):
MASRPAASSPVEARAPVGGQEAGGPSAATQGEAAGAPLAHGHHVYCQRVNGVMVLSDK
TPGSASYRISDSNFVQCGSNCTMIIDGDVVRGRPQDPGAAASPAPFVAVTNIGAGSDGGTA
VVAFGGTPRRSAGTSTGTQTADVPTEALGGPPPPPRFTLGGGCCSCRDTRRRSAVFGGEGD
PVGPAEFVSDDRSSDSDSDDSEDTDSETLSHASSDVSGGATYDDALDSDSSSDDSLQIDG
PVCRPWSNDTAPLDVCPGTPGPGADAGGPSAVDPHAPTPEAGAGLAADPAVARDDAEGL
SDPRPRLGTGTAYPVPLELTPENAEAVARFLGDAVNREPALMLEYFCRCAREETKRVPPRTF
GSPPRLTEDDFGLLNYALVEMQRLCLDVPPVPPNAYMPYYLREYVTRLVNGFKPLVSRSA

RLYRILGVLVHLRIRTREASFEEWLRSKEVALDFGLTERLREHEAQLVILAQALDHYDCLIH
STPHTLVERGLQSALKYEEFYLKRFGGHYMESVFQMYTRIAGFLACRATRGMRHIALGRE
GSWWEMFKFFFHRLYDHQIVPSTPAMLNLGTRNYYTSSCYLVNPQATTNKATLRAITSNV
SAILARNGGIGLCVQAFNDSGPGTASVMPALKVLDSLVAAHNKESARPTGACVYLEPWHT
DVRAVLRMKGVLAGEEAQRCDNIFSALWMPDLFFKRLIRHLDGEKNVTWTLFDRDTSMS
LADFHGEEFEKLYQHLEVMGFGEQIPIQELAYGIVRSAATTGSPFVMFKDAVNRHYIYDTQ
GAAIAGSNLCTEIVHPASKRSSGVCNLGSVNLARCVSRQTFDFGRLRDAVQACVLMVNIM
IDSTLQPTPQCTRGNDNLRSMGIGMQGLHTACLKLGLDLESAEFQDLNKHIAEVMLLSAM
KTSNALCVRGARPFNHFKRSMYRAGRFHWERFPDARPRYEGEWEMLRQSMMKHGLRN
SQFVALMPTAASAQISDVSEGFAPLFTNLFSKVTRDGETLRPNTLLLKELERTFSGKRLLEV
MDSLDAKQWSVAQALPCLEPTHPLRRFKTAFDYDQKLLIDLCADRAPYVDHSQSMTLYV
TEKADGTLPASTLVRLLVHAYKRGLKTGMYYCKVRKATNSGVFGGDDNIVCMSCAL

Ade6 (EV71-2C):
MSASWLKKFNDMANAAKGLEWISSKISKFIDWLKEKIIPAAREKVEFLNNLKQLPLLENQI
SNLEQSAASQEDLEAMFGNVSYLAHFCRKFQPLYATEAKRVYALEKRMNNYMQFKSKHR
IEPVCLIIRGSPGTGKSLATGIIARAIADKYHSSVYSLPPDPDHFDGYKQQVVMVMDDLCQ
NPDGKDISLFCQMVSTVDFIPPMASLEEKGVSFTSKFVIASTNSSNIIVPTVSDSDAIRRRFY
MDCDIEVTDSYKTDLGRLDAGRAAKLCSENNTANFKRCSPLVCGKAIQLRDRKSKVRYS
VDTVVSELIREYNNRSAIGNTIEALFQ

Ade7 (HBV-HBx):
MAARLCCQLDPARDVLCLRPVGAESRGRPLAGPLGALPPASPPVLPTDHGAHLSLRGLPV
CAFSSAGPCALRFTSARRMETTVNAHRNLPKVLHKRTLGLSAMSTTDLEAYFKDCVFNE
WEELGEEVRLKVFVLGGCRHKLVCSPAPCNFFTSA

AcrIIC1:
MANKTYKIGKNAGYDGCGLCLAAISENEAIKVKYLRDICPDYDGDDKAEDWLRWGTDS
RVKAAALEMEQYAYTSVGMASCWEFVEL

AcrIIA5:
MAYGKSRYNSYRKRSFNRSNKQRREYAQEMDRLEKAFENLDGWYLSSMKDSAYKDFGK
YEIRLSNHSADNKYHDLENGRLIVNIKASKLNFVDIIENKLDKIIEKIDKLDLDKYRFINATN
LEHDIKCYYKGFKTKKEVI

Raw Images for Supplementary Figure 2

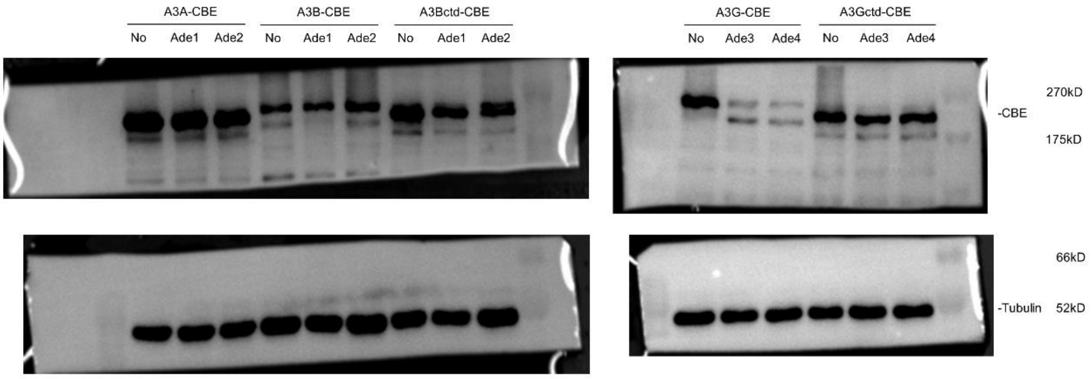

Raw Images for Supplementary Figure 5a

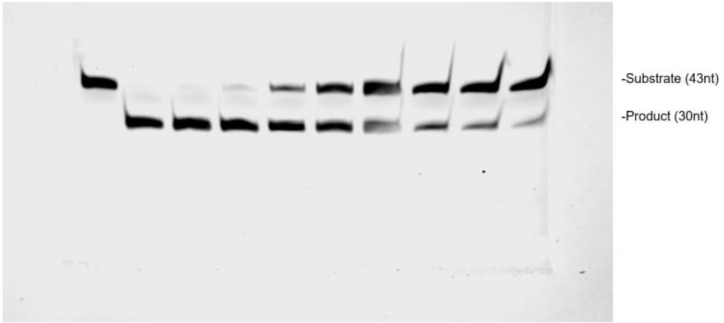

Raw Images for Supplementary Figure 5b

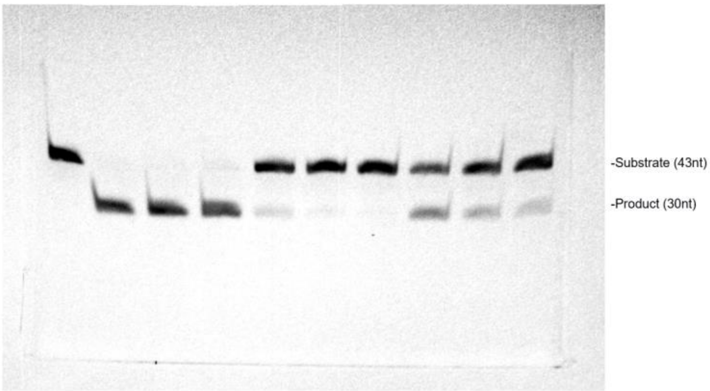

Supplement: Supplementary file 2 — Supplementary Information [file 41467_2022_28300_MOESM2_ESM.pdf]
