# Supplementary material for: Inhibition of base editors with anti-deaminases derived from viruses
Source: Nat Commun. 2022 Feb 1;13:597. doi: 10.1038/s41467-022-28300-0 (PMC8807840; doi:10.1038/s41467-022-28300-0)
Supplement: Supplementary file 3 — Reporting Summary [file 41467_2022_28300_MOESM3_ESM.pdf]

nature portfolio

Corresponding author(s): Zhanjun Li

Last updated by author(s): Jan 15, 2022

Reporting Summary

Nature Portfolio wishes to improve the reproducibility of the work that we publish. This form provides structure for consistency and transparency in reporting. For further information on Nature Portfolio policies, see our Editorial Policies and the Editorial Policy Checklist.

Statistics

For all statistical analyses, confirm that the following items are present in the figure legend, table legend, main text, or Methods section.

n/a	Confirmed	
☐	☒	The exact sample size (n) for each experimental group/condition, given as a discrete number and unit of measurement
☐	☒	A statement on whether measurements were taken from distinct samples or whether the same sample was measured repeatedly
☐	☒	The statistical test(s) used AND whether they are one- or two-sided<br>*Only common tests should be described solely by name; describe more complex techniques in the Methods section.*
☒	☐	A description of all covariates tested
☒	☐	A description of any assumptions or corrections, such as tests of normality and adjustment for multiple comparisons
☒	☐	A full description of the statistical parameters including central tendency (e.g. means) or other basic estimates (e.g. regression coefficient) AND variation (e.g. standard deviation) or associated estimates of uncertainty (e.g. confidence intervals)
☒	☐	For null hypothesis testing, the test statistic (e.g. F, t, r) with confidence intervals, effect sizes, degrees of freedom and P value noted<br>*Give P values as exact values whenever suitable.*
☒	☐	For Bayesian analysis, information on the choice of priors and Markov chain Monte Carlo settings
☒	☐	For hierarchical and complex designs, identification of the appropriate level for tests and full reporting of outcomes
☒	☐	Estimates of effect sizes (e.g. Cohen's d, Pearson's r), indicating how they were calculated

Our web collection on statistics for biologists contains articles on many of the points above.

Software and code

Policy information about availability of computer code

Data collection	The base editing efficiencies were quantified by EditR 1.0.10 (https://moriaritylab.shinyapps.io/editr_v10).
Data analysis	The data were analysed by two-sided t test via GraphPad Prism software 8.0.1. A probability value smaller than 0.05 (p < 0.05) was considered to be statistically significant. *p < 0.05, **p < 0.01, ***p < 0.001, ****p < 0.0001.

For manuscripts utilizing custom algorithms or software that are central to the research but not yet described in published literature, software must be made available to editors and reviewers. We strongly encourage code deposition in a community repository (e.g. GitHub). See the Nature Portfolio guidelines for submitting code & software for further information.

Data

Policy information about availability of data

All manuscripts must include a data availability statement. This statement should provide the following information, where applicable:

- Accession codes, unique identifiers, or web links for publicly available datasets
- A description of any restrictions on data availability
- For clinical datasets or third party data, please ensure that the statement adheres to our policy

All data generated or analysed during this study are included in this published article and its supplementary files. Source data are provided with this paper.

Field-specific reporting

Please select the one below that is the best fit for your research. If you are not sure, read the appropriate sections before making your selection.

☒ Life sciences ☐ Behavioural & social sciences ☐ Ecological, evolutionary & environmental sciences

For a reference copy of the document with all sections, see nature.com/documents/nr-reporting-summary-flat.pdf

Life sciences study design

All studies must disclose on these points even when the disclosure is negative.

Sample size	The experiments were repeated three independent biological replicates. These sample sizes were selected based on literature that used similar sample sizes to obtain gene editing results.
Data exclusions	No data weas excluded.
Replication	Values and error bars reflect the mean ± s.e.m. of three independent biological replicates. All attempts at replication were successful.
Randomization	Due to the small sample, randomization was not applicable for this study.
Blinding	Blinding was not relevant to our study. In general, based on the prior experience of other groups in the field, these types of assays do not require blinding.

Reporting for specific materials, systems and methods

We require information from authors about some types of materials, experimental systems and methods used in many studies. Here, indicate whether each material, system or method listed is relevant to your study. If you are not sure if a list item applies to your research, read the appropriate section before selecting a response.

Materials & experimental systems

n/a	Involved in the study	
☐	☒	Antibodies
☐	☒	Eukaryotic cell lines
☒	☐	Palaeontology and archaeology
☒	☐	Animals and other organisms
☒	☐	Human research participants
☒	☐	Clinical data
☒	☐	Dual use research of concern

Methods

n/a	Involved in the study	
☒	☐	ChIP-seq
☒	☐	Flow cytometry
☒	☐	MRI-based neuroimaging

Antibodies

Antibodies used	The antibody against Cas9 (1:1500; ab204448, Abcam) was used as a primary antibody, while Tubulin antibody (1:2000; 10094-1-AP, Wuhan Sanying) was used as the loading control.
Validation	The Cas9 antibody has been validated in many publications. eg:<br>Yu W et al. Harnessing A3G for efficient and selective C-to-T conversion at C-rich sequences. BMC Biol 19:34 (2021). Application: WB. Species: Human.<br>Liu Z et al. Precise base editing with CC context-specificity using engineered human APOBEC3G-nCas9 fusions. BMC Biol 18:111 (2020). Application: WB. Species: Human.<br>Martin AS et al. A panel of eGFP reporters for single base editing by APOBEC-Cas9 editosome complexes. Sci Rep 9:497 (2019). Application: WB. Species: Human.<br>St Martin A et al. A fluorescent reporter for quantification and enrichment of DNA editing by APOBEC-Cas9 or cleavage by Cas9 in living cells. Nucleic Acids Res (2018). Application: WB. Species: Human.<br>Wang X et al. Efficient base editing in methylated regions with a human APOBEC3A-Cas9 fusion. Nat Biotechnol N/A:N/A (2018). Application: WB. Species: Human.

Eukaryotic cell lines

Policy information about cell lines

Cell line source(s)	HEK293T (ATCC) and Huh7 (ATCC)

Authentication	The cell lines were authenticated by supplier and not further authenticated after receipt.
Mycoplasma contamination	All cell lines tested negative for mycoplasma.
Commonly misidentified lines (See ICLAC register)	No commonly misidentified cell lines were used in this study.
